# Optimising Fruit Harvesting Paths: A Mapless, Occlusion-Aware Picking Framework

**DOI:** 10.3390/s26123944

**Published:** 2026-06-21

**Authors:** Xuesong Ren, Yubin Miao

**Affiliations:** School of Mechanical Engineering, Shanghai Jiao Tong University, Shanghai 200240, China; renxuesong@sjtu.edu.cn

**Keywords:** viewpoint planning, mapless navigation, multi-task learning, picking guidance

## Abstract

Fruit harvesting is labor-intensive and increasingly challenged by the shortage of agricultural labor. To address viewpoint planning under occlusion, this paper proposes a mapless picking guidance framework that directly predicts the next viewing direction and estimates fruit occlusion without relying on pre-built maps or candidate-viewpoint sampling. Unlike conventional active vision methods that enumerate and evaluate multiple candidate viewpoints, the proposed method generates feasible viewpoints by jointly leveraging occlusion estimation and global picking direction supervision, thereby reducing computational cost and alleviating local optimum bias. An adaptive approach strategy is further introduced to balance viewpoint exploration and target approach during planning. Simulation results show that the proposed method achieves average success rates of 80.46% on the in-distribution test set and 77.58% on the unseen-fruit test set, with corresponding occlusion reductions of 80.56% and 79.16%, respectively. These results demonstrate the effectiveness of the proposed framework for occlusion-aware fruit viewpoint planning in unstructured orchard environments.

## 1. Introduction

Fruit harvesting is a labor-intensive and time-consuming process. With the continuous decline of agricultural labor supply, the cost associated with manual harvesting is increasing rapidly, leading to an urgent need for automation and robotization in agriculture [1]. The primary challenge for harvesting robots lies in accurately localizing the fruit in 3D space and planning an appropriate grasping trajectory. In practical orchard environments, fruits are frequently occluded by leaves and branches, which further complicates visual perception and motion planning.

Harvest failures commonly stem from poor picking pose estimation. A poor approaching direction makes the robotic end-effector interact inadvertently with branches or fruits. Then three major adverse outcomes often occur: (1) excessive fruit oscillation, which increases recognition difficulty and prolongs harvesting time; (2) significant displacement or tracking loss of the fruit; and (3) physical damage or fruit drop during the grasping process.

To address the challenges arising from occlusions, numerous studies have proposed vision-guided strategies for autonomous fruit harvesting. From the perspective of balancing exploration and exploitation, existing approaches can be broadly categorized into three groups.

The first group of methods relies on estimating the 3D pose of the fruit to provide reference information for robotic grasping orientation. These approaches typically require local 3D reconstruction of the environment surrounding the target fruit, from which the geometric pose of the fruit can be extracted to compute an optimal approach angle through explicit geometric reasoning. Some researchers have introduced shape completion techniques in either 3D [2] or 2D [3] domains, reconstructing occluded and partially visible fruits into complete shapes and estimating their pose for grasp point determination. Seo et al. built a point cloud dataset by pre-scanning apples of various sizes. After distinguishing fruits from background obstacles, they used a prior geometric model to infer position and orientation information to guide the grasp operation [4]. Li et al. utilized results from object detection and segmentation, introducing a 3D frustum-based approach to estimate the centroid of the fruit. By combining multiple stereo-vision views, the partially occluded fruit is reconstructed, and the best approach direction is estimated thereafter [5,6]. These methods aim to maximally exploit the available sensory information, which makes them computationally efficient and robust. However, the heavy reliance on local observations and deterministic reconstruction often leads to local optimality and limited adaptability to complex occlusions.

The second category encourages the robot to actively search rather than directly compute the optimal approach direction. Accordingly, these methods design search or exploration strategies that maintain a balance between exploration (seeking new informative viewpoints) and exploitation (using the current best estimate). Zhao et al. enabled the robot to move along fixed image-acquisition paths and select the minimally occluded viewpoint for harvesting [7]. Yi et al. adopted a randomized candidate view generation strategy and used the Spatial Coverage Rate Metric to evaluate the degree of occlusion, thereby guiding the adjustment of the approach angle [8]. Luo et al. developed an end-to-end active vision framework enhanced by deep reinforcement learning (DRL) for detecting grape stems under heavy occlusion. The information-gain-based reward function directs the manipulator toward the most informative viewpoints [9]. These approaches endow the robot with exploratory capabilities, helping to avoid local optima and improving robustness in cluttered environments. Nevertheless, DRL-based training is costly in terms of computation and time, and it generally requires precise environmental modeling. Moreover, the trained policy often exhibits limited generalization across different orchard scenes.

The third type of method neither focuses on fruit pose estimation nor on designing explicit exploration policies. Instead, it performs direct path search and optimization based on full 3D reconstruction of the local environment, aiming for nearly global-optimal harvesting trajectories. Gao et al. modeled apples and branches as spheres and cuboids with collision volumes, respectively, and improved a particle swarm optimization (PSO) algorithm for harvesting path planning [10]. Similarly, Xu et al. modeled three-dimensional obstacles in occluded scenes and applied the Enhanced Space Tree (EST) algorithm to perform obstacle-avoidance path planning [11]. Although these methods rely on complete spatial perception and high computational load, they generally guarantee near-optimal solutions in terms of collision-free path generation and harvesting efficiency.

Existing active vision-based methods generally follow a candidate-viewpoint selection paradigm, in which the next observation point is chosen from a set of viewpoints according to a predefined scoring strategy. Although effective in simple cases, such a discrete selection scheme is prone to local optimality, particularly in cluttered orchard environments with severe occlusions. To overcome this limitation, we propose a novel Mapless Picking Guidance Framework with the following contributions.

1. We take the globally optimal picking viewpoint as supervision and train an end-to-end guidance model to directly predict the approaching direction, thereby avoiding the tendency of candidate-based planning to become trapped in local optima.
2. We further design a multi-task model that reuses the same visual features for both direction guidance and occlusion rate prediction, which substantially improves computational efficiency.
3. We introduce an adaptive approaching strategy driven by the predicted occlusion level. This strategy encourages the camera to move closer to the target fruit when occlusion is low, while promoting further viewpoint exploration when the target is heavily occluded, thus achieving a more effective balance between exploration and exploitation.

## 2. Materials and Methods

### 2.1. Simulation Environment

**Figure 1:**
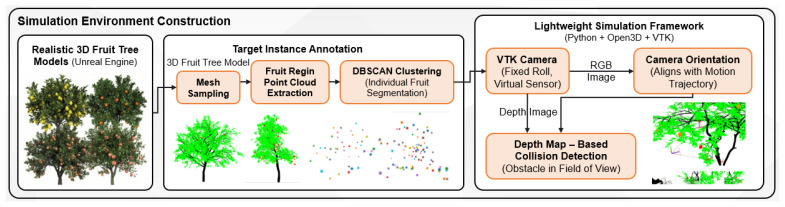
Simulation Environment Framework.

In computer simulation of fruit-picking or path-planning tasks, the 3D fruit tree models are often overly simplified compared with the densely branched and leafy trees in the real world. To obtain more realistic rendered images, we employed tree models from the Unreal Engine and constructed a lightweight simulation environment inspired by the framework of UnrealCV [12]. As shown in Figure 1, the algorithmic environment of this work was implemented with Python 3.10, utilizing Open3D 0.19.0 and VTK 9.5.2 libraries, and executed on a workstation equipped with an NVIDIA GeForce RTX 3080 GPU(NVIDIA Corporation, Santa Clara, CA, USA). Since our focus is on visual algorithms, we did not build a physical robot or manipulator. A VTK camera with a fixed roll angle was used to simulate sensor outputs, capturing RGB and depth images from specified viewpoints. Whenever the camera moves, its viewing direction is adjusted to align with the movement trajectory, after which a depth map is captured. In this way, obstacle perception is achieved without relying on complex collision-detection procedures. Meanwhile, the trajectory of the VTK camera could be recorded and visualized to analyze camera motion paths.

**Figure 2:**
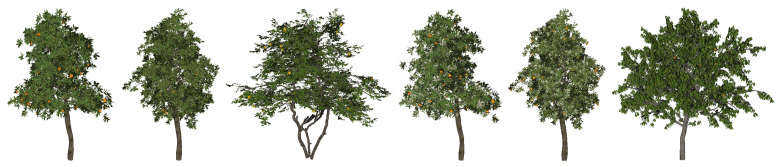
3D mesh models of fruit trees used in the simulation environment.

We collected six three-dimensional fruit tree models, including lemon, orange, and peach trees, with fruits attached, as shown in Figure 2. We sampled the fruit regions from the mesh models and converted them into point clouds. The DBSCAN clustering algorithm was then employed to segment and identify individual fruit instances.

### 2.2. Global Optimal Picking Angle as Supervision

#### 2.2.1. Mathematical Definition of the Optimal Picking Angle

We employ a ray-based model to describe the occlusion relationship between the picking target and its surrounding environment from a given viewpoint. Let the centroid of the target object *O* be located at the origin of the coordinate system. From any point *o* on *O*, a ray directed toward the observer’s position is denoted as $\vec{ob}$. Assuming that this ray continues to extend beyond the observer, for an unobstructed ray we have $∥\vec{ob}∥\to +\infty$, whereas for a self-occluded internal point $∥\vec{ob}∥\to 0$. Accordingly, for a given viewpoint, the occlusion ratio of the object can be expressed as:

(1) $$occ_{b}=\frac{\left|\{\vec{ob}|o\in O,\epsilon <∥\vec{ob}∥<+\infty \}\right|}{\left|\{\vec{ob}|o\in O,∥\vec{ob}∥>\epsilon \}\right|}.$$

If we define the observation distance to the target centroid as *z*, then the observation points b∉O are distributed on a spherical surface $B_{r=z}$. Without considering any change in the pose of the picking target *O*, the grasping action can be simplified as a translation of *O* along the radial direction ${\vec{r}}_{b}$ that passes through the spherical surface *B*. In this case, the motion trajectories of all points on *O* can be represented as:

(2) $$R_{b}=\{\vec{o^{\prime }b^{\prime }}|\vec{o^{\prime }b^{\prime }}\left\|{\vec{r}}_{b},b^{\prime }\in B,o^{\prime }\in O\right\}.$$

The set of all $b^{\prime }$ can be regarded as the projection of *O* onto the spherical surface *B* along the current radial direction ${\vec{r}}_{b}$ of the observation point. Based on this set of motion trajectories, we can determine whether the observation point *b* is a feasible picking viewpoint that allows a collision-free grasping operation. All feasible picking points can be defined as:

(3) $$P=\{b|\forall \vec{o^{\prime }b^{\prime }}\in R_{b},∥\vec{o^{\prime }b^{\prime }}∥\to +\infty \}.$$

For an observation point *b*, the optimal picking direction $\vec{r_{p}}$ can then be defined as the direction from the target centroid toward the nearest feasible picking point p∗ on the spherical surface:

(4) $$p*=\underset{p\in P}{\arg\min}∥\vec{pb}∥.$$

#### 2.2.2. Redesign of Training Labels

**Figure 3:**
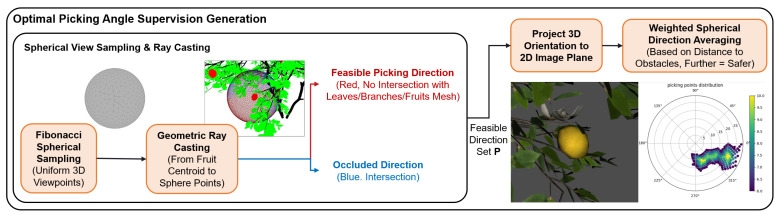
(**Left**): Application of a Fibonacci lattice for sampling viewpoints around the target fruit. In the texture-free fruit tree model, fruits, leaves, and trunk are shown in red, green, and black, respectively. Feasible picking directions on the lattice are highlighted in red, while occluded viewpoints are indicated in blue. (**Right**): Polar coordinate representation of the projection distribution of feasible picking points near the target fruit onto the camera image plane.

We employ a Fibonacci lattice to uniformly sample viewpoints on the spherical surface $B_{r=1}$, thereby discretizing the observation directions. As illustrated in Figure 3, on the unit sphere, 2000 rays $\vec{ob}$ are generated by connecting the centroid of the target fruit *O* to each sampled point on the sphere. For rays satisfying $∥\vec{ob}∥\to +\infty$, that is, those not intersecting with any triangular facet of the fruit tree model, red color is assigned. Conversely, rays that intersect any facet are marked in blue.

We represent the Fibonacci lattice as an undirected graph, assigning a value to each vertex to indicate its distance to the nearest feasible picking point. In the graph, each vertex represents a viewpoint, and the edges connect adjacent viewpoints on the Fibonacci lattice. Red and blue indicate different attributes. Starting from any blue vertex, we use Dijkstra’s algorithm to compute the shortest path in the undirected graph to any red vertex. All feasible picking points form a continuous region on the lattice surface, and any grasping or picking action approaching this region should be successful.

When the target fruit is occluded, it is difficult to directly infer the optimal 3d picking direction from the RGB images captured by the vision sensor mounted at the end of the robotic manipulator. This difficulty arises not only from the loss of depth information but also because many obstacles themselves are occluded. To reduce the learning complexity of the model, we redesign the action space of the visual sensor. The camera is allowed to adjust its viewpoint to face the visible region of the grasping target while maintaining a fixed roll angle. The camera can also choose to move closer to or farther from the target, or to translate within the image plane by a 2d direction $\vec{d}=\left(d_{x},d_{y}\right)$. However, this direction cannot be simply determined as the projection, on the image plane, of the direction toward the nearest feasible picking point from the current observation point.

Figure 3 also shows the relative spatial distribution of all feasible picking points with respect to the current viewpoint. The value assigned to each picking point is proportional to its distance from the nearest obstacle. Therefore, higher values (shown in yellow) correspond to picking angles that are less likely to cause collisions. Intuitively, moving downward and to the right increases the likelihood of a successful pick. However, the nearest feasible picking point lies directly below the current viewpoint. We reformulate the supervisory signal into a weighted form:

(5) $$\vec{d}=\mathrm{PROJ}\frac{\sum_{p\in P}\left(1-\frac{∥\vec{op}{∥}^{2}}{\max_{p^{\prime }\in P}{∥\vec{op^{\prime }}∥}^{2}}\right)\vec{op}}{\sum_{p\in P}\left(1-\frac{∥\vec{op}{∥}^{2}}{\max_{p^{\prime }\in P}{∥\vec{op^{\prime }}∥}^{2}}\right)}.$$

We optimize computations by exploiting the distances between grid points on the Fibonacci lattice. This weighting process guides the final decision toward an orientation that is relatively close but safer. At this stage, the viewpoint guiding model no longer directly outputs the optimal 3d picking direction; instead, it indicates the 2d direction of the optimal picking angle relative to the current viewpoint in the image plane.

### 2.3. Design and Evaluation of the Mapless Picking Guidance Module

#### 2.3.1. Dual-Task Learning-Based Direction Predictor and Occlusion Ratio Proxy

**Figure 4:**
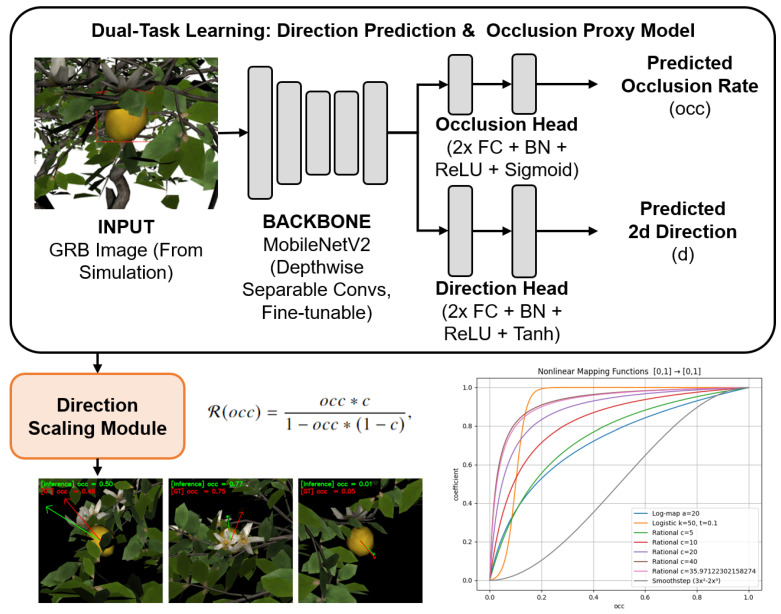
Mapless Picking Guidance Framework.

We developed a simple neural network model based on multi-task learning, as shown in Figure 4. The network takes an RGB image captured from a given viewpoint *b* as input and outputs two quantities: (1) occ, the estimated percentage of the fruit area occluded by leaves or branches, and (2) $\vec{d}=\left(d_{x},d_{y}\right)$, a 2D direction vector indicating the suggested camera movement for obtaining a less occlusion next view.

Our deep learning model consists of an interchangeable backbone and two distinct prediction heads. The feature extraction backbone employs a pre-trained MobileNetV2 architecture, utilizing its depth-wise separable convolutions for computational efficiency. The feature extractor consists of 18 convolutional layers, with the final layer producing feature maps with 1280 channels. To balance transfer learning benefits and adaptation to our specific task, we freeze all but the final convolutional block during initial training phases. Occlusion Prediction Head is a classification sub-network that estimates the probability of visual occlusion. This branch employs two fully connected layers with batch normalization and ReLU activation, culminating in a sigmoid output that produces occlusion probabilities in the range [0, 1]. Direction Prediction Head is a regression sub-network that predicts 2D directional vectors. This branch uses a similar architecture to the occlusion head but employs tanh activation to constrain outputs to the range [−1, 1]. The final directional output is modulated by a rational mapping function R(occ) that scales the raw directional predictions based on the estimated occlusion probability:

(6) $$R\left(occ\right)=\frac{occ*c}{1-occ*(1-c)},$$

where the constant c=35.97 is determined such that the mapping function passes through the point (0.1, 0.8). When the occlusion ratio is less than 0.1, this scale coefficient rapidly decreases from 0.8 to 0; whereas when occ>0.1, the scale coefficient remains stable near 1. Our goal is to encourage the model to explore the surroundings more when occlusion is high, while moving quickly toward the target when occlusion is low. Specifically, exploration of the surroundings corresponds to a larger $\vec{d}$, while the forward and backward component dz is not dependent on this model. This decoupling reduces the learning complexity.

For this dual-task network model, we redesign the loss function as a weighted sum of three components:

(7) $$L_{\mathrm{total}}=\alpha \cdot L_{\mathrm{dir}}+\beta \cdot L_{\mathrm{mod}}+\gamma \cdot L_{\mathrm{occ}}=\alpha \cdot E\left[\Delta \theta^{2}\right]+\beta \cdot L_{\mathrm{mod}}+\gamma \cdot L_{\mathrm{occ}},$$

where,

(8) $$\Delta \theta =\left[(\arctan\left({\vec{d}}_{y}^{pred},{\vec{d}}_{x}^{pred}\right)-\arctan\left({\vec{d}}_{y}^{gt},{\vec{d}}_{x}^{gt}\right)+\pi )\;\mathrm{mod}\;2\pi \right]-\pi .$$

Directional Loss ($L_{\mathrm{dir}}$) measures the angular difference between predicted and ground truth directions. Occlusion Loss ($L_{\mathrm{occ}}$) applies mean squared error (MSE) on the occlusion probabilities. Modulus Loss ($L_{\mathrm{mod}}$) enforces consistency of the vector magnitude, also using MSE.

During training, we observe that the directional loss dominates the total loss, and that when occ is small, direction prediction should not be the primary focus of the model. Thus, we assign equal weights to occlusion loss and modulus loss, and set the directional loss weight to be one-twentieth of those.

#### 2.3.2. Adaptive Trajectory Generation

To generate the camera trajectory, we update the viewpoint incrementally based on the direction predicted by our model. Instead of explicitly introducing a separate obstacle avoidance module, the model directly guides the camera toward a more open and less occluded region, which implicitly reduces the likelihood of moving into highly obstructed areas.

(9) $$d_{z}=\lambda \cdot \left(1-occ\right)\cdot \max(0,1-|d_{x}\left|-\right|d_{y}\left|\right),$$

where λ∈[0,1] is a scaling factor used to regulate how aggressively the camera moves toward the target.

To adapt the motion according to the predicted visibility of the target, we set

(10) λ=1−occ,

When the target is heavily occluded, the forward motion is suppressed so that the camera can continue exploring more informative viewpoints in the surrounding free space. As the occlusion level decreases, the camera is allowed to approach the target more closely. In this way, the trajectory is driven by a visibility-aware viewpoint policy rather than by an explicit obstacle avoidance mechanism.

#### 2.3.3. Evaluation of the Mapless Picking Guidance Model

**Figure 5:**
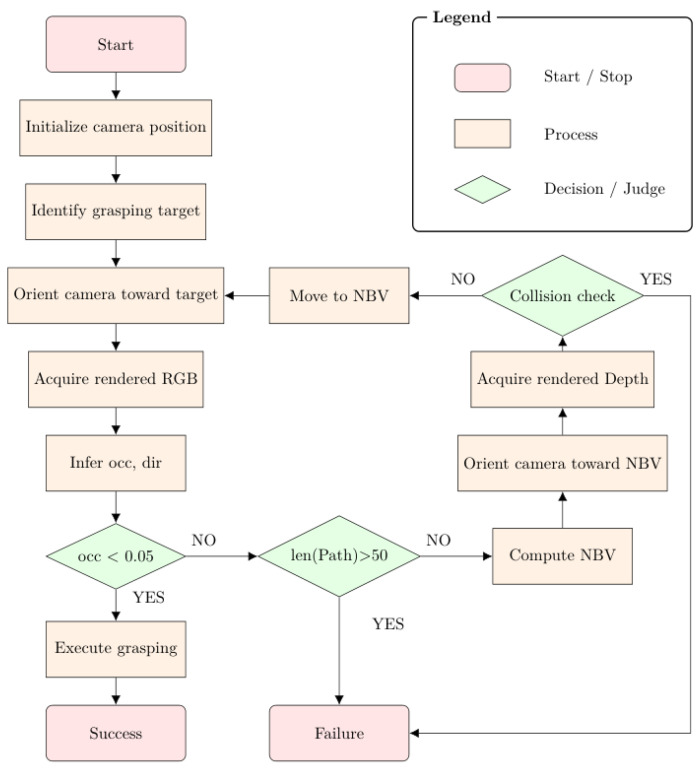
Procedure flowchart for picking task evaluation using mapless guidance model.

Figure 5 presents the evaluation pipeline of the grasping task under the proposed mapless picking guidance framework. For each target fruit, 2000 initial camera positions $b_{0}$ are sampled around the fruit using a Fibonacci lattice. From each initial viewpoint, a picking simulation is carried out and the outcome is recorded as success or failure. The Viewpoint Planning Success Rate is then computed from all trials. We further measure the occlusion reduction between the initial and final viewpoints to quantify the improvement in target visibility achieved during planning.

The 32 test fruits are divided into two groups, namely TEST 1 and TEST 2, with 16 fruits in each group. TEST 1 contains fruits that provide several training samples for the Direction Predictor, whereas TEST 2 consists of unseen fruits, including samples from different trees, and is used to evaluate the generalization ability of the proposed method under more challenging conditions.

During simulation, the agent iteratively observes the target fruit, predicts the approaching direction, moves accordingly, and reorients toward the fruit from the new position. No explicit collision-avoidance module is used; instead, any collision with branches or leaves is regarded as a failure, ensuring that viewpoint improvement is driven solely by the proposed guidance model. To avoid ineffective oscillations around local optima, a simulation is also terminated as a failure if the predicted occlusion $\hat{occ}$ does not decrease for 10 consecutive steps or if the total number of steps exceeds 50. Since the predicted occlusion may be affected by mesh penetration and occlusion approximation errors, the final graspability is determined using the actual occlusion at the final viewpoint. A viewpoint is considered directly pickable if occ<0.05.

## 3. Results

### 3.1. Fruit Instance Segmentation

**Figure 6:**
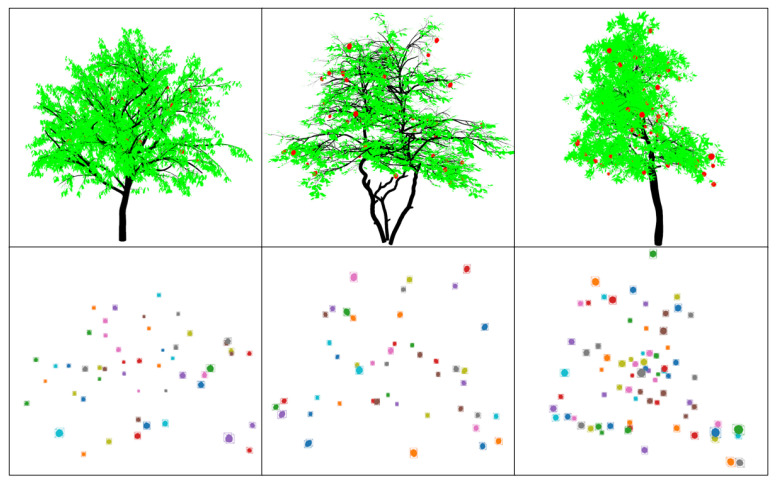
Distribution of fruit instance segmentation results for one lemon tree, one orange tree, and one peach tree.

By clustering the point cloud sampled from the original 3D model, we performed instance segmentation of individual fruits and computed their minimum bounding boxes, as illustrated in Figure 6. We then reconstructed the surfaces and converted the point clouds back into meshes, enabling editing of single fruits within the simulation environment.

### 3.2. Dual-Task Learning

**Figure 7:**
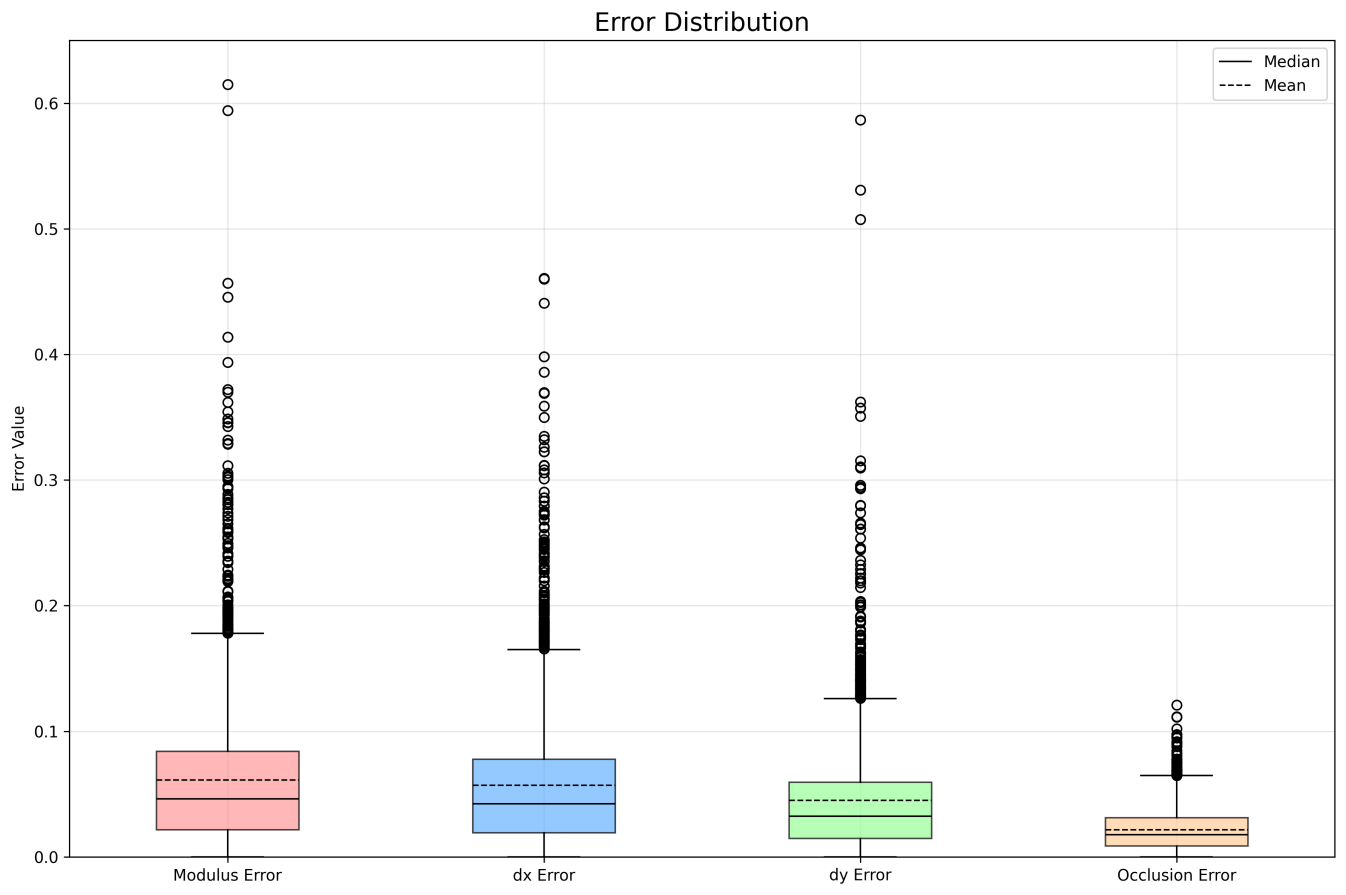
Boxplots of errors for 2D direction vector and occlusion ratio on test dataset.

We selected 40 fruit targets from six fruit tree models and randomly sampled viewpoints around each fruit. Using the method described above, we generated the optimal viewpoint direction and occlusion rate for each viewpoint as supervision signals. The resulting training set contains 16,000 samples. After splitting the dataset into training and testing sets in an 8:2 ratio, we further applied random cropping and random rotation for data augmentation on the training set. As shown in Figure 7, on the test set, the model achieves a magnitude error of 0.0611±0.0578, with directional component errors of 0.0570±0.0539 along the *x*-axis and 0.0450±0.0469 along the y-axis. The occlusion error is 0.0216±0.0171.

**Figure 8:**
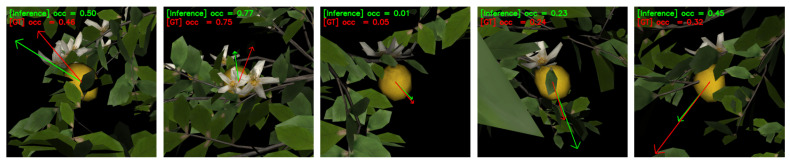
Inference results of the dual-task learning model. During training and testing, the RGB images were preprocessed by setting the background to (0, 0, 0); hence, the background appears black.

We overlaid the rendered viewpoint images with the predicted directions, observing that the predictions generally point toward more open areas surrounding the fruit, as shown in Figure 8. The red arrows indicate the projected directions of the optimal picking angles computed using the Fibonacci lattice, while the blue arrows represent the directions guided by the model. The length of each arrow also reflects the distance from the current viewpoint to the optimal picking direction. In the figure, all arrow lengths are scaled by a factor of 500 for better visualization.

Occasionally, there are significant angular deviations, which could be critical shortcomings for the model. However, since we do not rely on a single image to predict the optimal picking angle but rather iteratively approach it, such deviations are acceptable. Notably, larger directional errors tend to coincide with higher occlusion levels, which is reasonable because under strong occlusion, the model tends to explore alternative viewpoints rather than approach the target directly. Although this exploration may involve longer paths, once occlusion decreases, the model can predict more accurate directions that rapidly guide the viewpoint toward the optimal picking direction. This behavior aligns well with our expectations.

### 3.3. Mapless Picking Guidance

**Figure 9:**
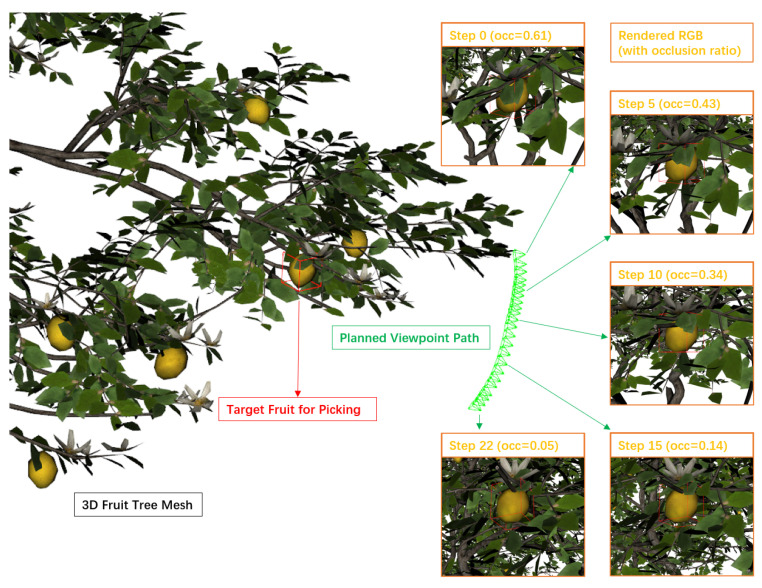
An example of a planned viewpoint path. In the final camera view, the degree of occlusion of the target fruit was reduced from 0.61 to 0.05.

We classify the target fruit’s occlusion levels at each viewpoint into three categories based on occlusion rates: mild occlusion (0.05–0.35), moderate occlusion (0.35–0.65), and severe occlusion (0.65–0.95). Conversely, viewpoints with occlusion rates above 0.95 are deemed nearly impossible for accurate fruit recognition; thus, no picking attempts are made from such viewpoints. Additionally, as initial viewpoints are randomly generated near the target fruit, some may be located inside branches or leaves. During camera movement attempts, we discard any initial viewpoints that experience collisions with branches or leaves within five steps. For the remaining viewpoints, we apply the Mapless Picking Guidance Model for viewpoint planning, and an example result is presented in Figure 9.

A collision during viewpoint change is considered a failure. If the occlusion level of the target fruit does not decrease after ten consecutive steps, the attempt is also deemed a failure. Moreover, if after fifty steps the occlusion level remains above 0.05, the attempt is considered unsuccessful. Viewpoints with occlusion rates below 0.05 are regarded as directly pickable, meaning that once the planned viewpoint reaches this condition, no further exploration for better picking angles is required and the corresponding picking operation can be executed immediately. Using these criteria, we record and statistically analyze the success rates starting from random viewpoints at different occlusion levels for each fruit. As previously described, the test fruits are split into two groups: TEST 1, which includes fruits providing several training samples to the model, and TEST 2, which consists of fruits unseen during training, including some from different fruit trees.

We implemented a candidate-viewpoint-based method (CVB) as a baseline for comparison. Existing active vision approaches typically perform viewpoint rendering based on local 3D reconstruction and then select the best viewpoint from a set of candidates according to a predefined strategy as the next observation point. To make a fair comparison, we added a viewpoint sampling module in the simulation environment, where six candidate viewpoints are sampled around the current viewpoint, including front, back, left, right, up and down. Among these candidates, the viewpoint with the lowest ground truth fruit occlusion rate is selected as the next viewpoint. The same evaluation protocol is used to record whether the viewpoint planning simulation succeeds or fails.

For the 32 target fruits included in the two test sets, we randomly sampled initial viewpoints and conducted simulations, resulting in a total of 35,918 viewpoint planning trials. As shown in Table 1, the proposed method consistently outperforms the candidate-viewpoint-based baseline (CVB) on both TEST 1 and TEST 2, particularly under medium and severe occlusion conditions. On TEST 1, our method achieves an overall success rate of 0.8046, which is substantially higher than the 0.6158 obtained by CVB. A similar improvement is observed on TEST 2, where the success rate increases from 0.6263 to 0.7758, demonstrating that the proposed approach not only performs well on fruits with training samples but also generalizes effectively to unseen fruits, including those from different trees. In addition, the advantage of our method becomes more pronounced as the occlusion level increases. While the success rate of CVB drops sharply under severe occlusion, our method maintains relatively strong performance, achieving 0.6202 on TEST 1 and 0.5844 on TEST 2. This indicates that the proposed guidance model is capable of locating reachable viewpoints even in highly cluttered environments.

Table 2 further confirms the superiority of the proposed method in reducing target-fruit occlusion. Across both test sets and all occlusion categories, our method consistently achieves higher occlusion reduction rates than CVB. On TEST 1, the overall occlusion reduction rate reaches 0.8056, compared with 0.6562 for CVB; on TEST 2, the corresponding values are 0.7916 and 0.6540, respectively. In particular, the proposed method shows strong performance under severe occlusion, where it still yields substantial occlusion reduction, highlighting its robustness in challenging scenarios. By contrast, CVB exhibits a noticeable performance degradation when the initial viewpoint is heavily occluded, suggesting that its greedy candidate-selection strategy is less effective in complex branch-and-leaf environments.

Overall, these results demonstrate that the proposed Mapless Picking Guidance Model can not only improve viewpoint planning success but also more effectively reduce fruit occlusion during the navigation process. Importantly, these gains are observed on both seen and unseen fruits, indicating strong generalization ability. The results also suggest that continuous, mapless viewpoint guidance provides a more reliable strategy than discrete candidate-viewpoint selection for fruit picking in cluttered orchard environments.

### 3.4. Trajectory Generation

**Figure 10:**
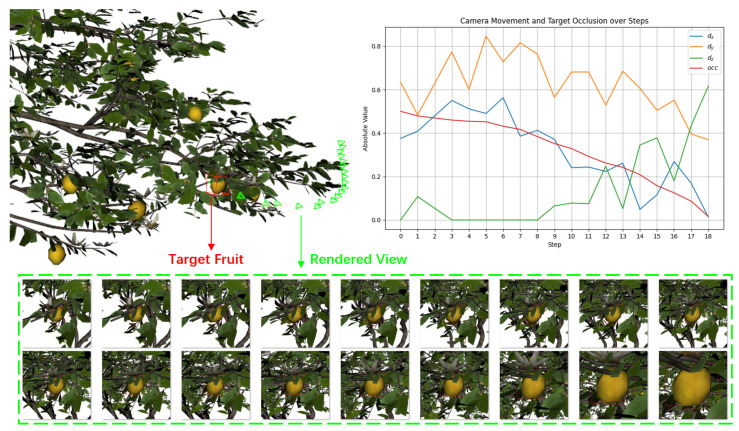
Example of a planned viewpoint path with adaptive scaling factor λ. Scale λ is used to control whether the trajectory approaches the target faster or more slowly. The value used in the figure is λ=1−occ.

Figure 10 presents a representative successful trajectory generated by the proposed adaptive viewpoint planning strategy. The camera initially performs lateral exploration in the free space and subsequently moves closer to the target as the occlusion level decreases. This trajectory pattern confirms that the scaling factor λ=1−occ can effectively regulate the progression of the camera according to target visibility.

The adaptive mechanism is particularly beneficial in cluttered scenes. When the fruit is heavily occluded, suppressing the forward motion helps avoid premature convergence toward obstructed regions and encourages further exploration of informative viewpoints. Once the occlusion is reduced, the camera can advance more aggressively toward the target, thereby accelerating the transition to a directly pickable viewpoint. Such a visibility-aware motion policy provides a better balance between exploration and exploitation than a fixed-step strategy.

These observations indicate that the adaptive scaling factor is effective in improving planning stability and motion efficiency. By dynamically adjusting the forward displacement based on the predicted occlusion level, the proposed method can both prevent unnecessary motion under poor visibility and facilitate rapid convergence when a clearer view becomes available.

## 4. Discussion

This study establishes a comprehensive simulation environment and proposes a dual-task learning model for global optimal picking direction guidance and occlusion ratio estimation. Based on this model, we further develop a mapless picking guidance framework that supports picking-path planning and decouples viewpoint motion from explicit 3D reconstruction, while employing an adaptive approaching strategy to guide the camera toward the target fruit. Overall, the proposed framework provides a new perspective for fruit picking under complex occlusion conditions and offers a more complete formulation of the problem together with an objective and consistent evaluation protocol.

Despite these contributions, several limitations remain. First, the construction of the dataset is still constrained by the difficulty of obtaining high-quality 3D fruit tree models. Although such models are more controllable than real-world point cloud data, their acquisition remains labor-intensive and is therefore not yet scalable. In addition, the rendered images in our simulation environment rely on ground truth signals, whereas real-world RGB-D perception is inevitably affected by sensor noise and measurement errors. These differences suggest that sim-to-real transfer remains an important direction for future work.

A core idea of the proposed direction guidance model is that it does not directly regress the absolute optimal picking angle. Instead, it predicts the relative direction from the current viewpoint toward the region where the optimal picking angle is located. If this directional prediction were perfectly accurate, the manipulator following it would not miss the optimal region, although it might still reach an earlier feasible viewpoint. This behavior is desirable because a feasible viewpoint usually already provides sufficiently low occlusion and can be reached with fewer steps. In practice, however, the direction predictor cannot always guarantee perfect directional accuracy. Fortunately, the optimal solution is not a single isolated direction but rather a feasible viewing region within a cone, which makes the model more stable than a strictly point-based prediction. A remaining limitation is that the camera must autonomously reorient itself to face the target fruit. Whether the proposed framework can still provide reliable direction guidance when the fruit is not centered in the image has not yet been fully investigated. Moreover, we have not quantitatively analyzed how direction prediction accuracy changes when the camera is rotated away from the fruit center or aimed at the centroid of the visible fruit region. These issues are closely related and deserve further study toward a more general occlusion-aware navigation paradigm.

The feature extractor used in the direction prediction branch is a widely validated convolutional neural network backbone, and we do not explore alternative architectural designs in depth. As a result, there remains considerable room for improvement in both representation quality and prediction accuracy. Nevertheless, the proposed framework avoids explicit 3D reconstruction of the scene, which improves computational efficiency and deployment feasibility. At the same time, mapless guidance is still generally less robust than reconstruction-based approaches in highly dynamic or extremely cluttered environments, which remains one of the main obstacles to real-world deployment.

The strategy for selecting initial viewpoints in the picking simulation is also worth further discussion. In practical orchard operations, sensors are typically positioned outside the canopy rather than inside the tree, and therefore unrealistic viewpoints can be filtered by checking whether the predicted optimal direction is opposite to the current observation direction. This filter removes some invalid samples, but it cannot guarantee that all remaining viewpoints are fully appropriate. In addition, manually specified initial viewpoints may compromise objective evaluation, whereas purely random viewpoints do not necessarily match the distribution encountered in actual picking tasks. In real applications, the manipulator is usually located outside the canopy, where nearby feasible picking viewpoints are more likely to exist. Therefore, determining a realistic viewpoint distribution according to the robot configuration could reduce the burden on dataset construction and further improve model performance.

This study currently considers only single-target picking and does not address active scene exploration or multi-target optimization. While a serial execution strategy is straightforward, higher efficiency could potentially be achieved by extending the framework to handle multiple targets jointly. Fortunately, the simulation environment we developed allows target fruits to be easily edited or replaced, which provides a convenient basis for future extensions in this direction.

For occlusion-aware fruit picking, a comprehensive evaluation framework still requires further investigation. We believe that picking difficulty is jointly influenced by the initial viewpoint and the relative spatial configuration between the target fruit and its surrounding branches and leaves. Therefore, it is meaningful to define one or more objective indices to quantify picking difficulty. In this work, we use the occlusion ratio occ as a practical indicator for evaluating viewpoint difficulty under different observation conditions. The distribution characteristics of occ may serve as an objective measure of the difficulty of individual fruits, which could be useful for future benchmarking studies.

In this work, the occlusion threshold of 0.05 is used to determine whether a viewpoint planning process has reached a sufficiently good observation state, rather than to guarantee successful physical grasping. In our setting, this threshold mainly separates views that are only lightly occluded by the stem from those that are more heavily blocked by leaves or branches. Empirically, ten typical stem-occluded views have an average occlusion ratio of 0.0378, which supports the use of 0.05 as a practical cutoff. Moreover, a stopping threshold is necessary during planning; if it is too strict, the model may keep searching until the step limit is reached and be incorrectly marked as failed. Since the predicted occlusion is also subject to estimation error, the threshold should balance practical stopping behavior and predictor stability. A more rigorous statistical determination of this threshold is left for future work.

## Figures and Tables

**Table 1:** Viewpoint Planning Success Rates under Different Occlusion Levels on TEST 1 and TEST 2 with 95% Confidence Intervals.

Method	Overall	Mild Occlusion	Moderate Occlusion	Severe Occlusion
TEST 1				
CVB	0.6158	0.8986	0.6126	0.3694
	[0.5684, 0.6631]	[0.8480, 0.9491]	[0.5216, 0.7037]	[0.2937, 0.4452]
Ours	0.8046	0.9539	0.8132	0.6202
	[0.7983, 0.8109]	[0.9485, 0.9593]	[0.8020, 0.8243]	[0.6066, 0.6339]
TEST 2				
CVB	0.6263	0.8750	0.5304	0.3904
	[0.5990, 0.6537]	[0.8456, 0.9044]	[0.4777, 0.5832]	[0.3409, 0.4399]
Ours	0.7758	0.9457	0.7666	0.5844
	[0.7576, 0.7941]	[0.9295, 0.9619]	[0.7332, 0.8000]	[0.5462, 0.6226]

**Table 2:** Occlusion Reduction under Different Occlusion Levels on TEST 1 and TEST 2 with 95% Confidence Intervals.

Method	Overall	Mild Occlusion	Moderate Occlusion	Severe Occlusion
TEST 1				
CVB	0.6562	0.6566	0.7447	0.5933
	[0.6273, 0.6851]	[0.6196, 0.6935]	[0.6951, 0.7942]	[0.5377, 0.6489]
Ours	0.8056	0.8194	0.8572	0.7396
	[0.8008, 0.8105]	[0.8131, 0.8257]	[0.8490, 0.8655]	[0.7293, 0.7499]
TEST 2				
CVB	0.6540	0.6552	0.6831	0.6256
	[0.6380, 0.6700]	[0.6369, 0.6734]	[0.6504, 0.7159]	[0.5915, 0.6597]
Ours	0.7916	0.8366	0.8227	0.7086
	[0.7774, 0.8058]	[0.8188, 0.8543]	[0.7969, 0.8485]	[0.6789, 0.7383]

## Data Availability

The data and code presented in this study will be made openly available in GitHub at https://github.com/SmallSnowRXS/Mapless-Picking-Guidance-Framework, 11 June 2026.
